# *Streptococcus cristatus* bacteremia in a patient with poor oral hygiene: a case report

**DOI:** 10.1186/s13256-023-03818-z

**Published:** 2023-05-17

**Authors:** Camilo Guzman, Adi Zaclli, John Molinari

**Affiliations:** 1 Department of Psychiatry, Henry Ford Health System, 2799 W Grand Blvd., Detroit, MI 48202 USA; 2 Department of Internal Medicine, Detroit Medical Center, Detroit, MI USA; 3 Department of Internal Medicine, Henry Ford Health System, Detroit, MI USA

**Keywords:** *Streptococcus cristatus*, Bacteremia, Endocarditis, Cirrhosis, Cephalosporins

## Abstract

**Background:**

*Streptococcus cristatus* is a member of the Mitis streptococcus group. Like other members of this group, it resides on mucosal surfaces of the oral cavity. However, little is known about its ability to cause disease as there are only a handful of cases in the literature. Two of these cases involved infective endocarditis with significant complications. However, these cases involved additional microbes, limiting the inferences about the pathogenicity of *Streptococcus cristatus*.

**Case presentation:**

A 59-year-old African American male with end-stage cryptogenic cirrhosis and ascites presented with fatigue and confusion. A paracentesis was negative for spontaneous bacterial peritonitis, but two separate blood cultures grew *Streptococcus cristatus*. Our patient had a history of dental caries and poor oral hygiene, which were likely the source of the infection. Echocardiograms revealed new aortic regurgitation, indicating “possible endocarditis” per the Modified Duke Criteria. However, since his clinical picture and cardiac function were reassuring, we elected against treatment for infective endocarditis. He was treated for bacteremia with a 2-week course of cephalosporins consisting of 8 days of ceftriaxone, transitioning to cefpodoxime after discharge. Despite having end-stage liver disease, our patient did not experience any significant complications from the infection.

**Conclusion:**

A patient with end-stage cirrhosis and poor oral hygiene developed bacteremia with an oral bacterium called *Streptococcus cristatus*. Unlike previous cases in literature, our patient did not meet criteria for a definitive diagnosis of infective endocarditis, and he experienced no other complications from the infection. This suggests coinfectants may have been primarily responsible for the severe cardiac sequelae in prior cases, whereas isolated *Streptococcus cristatus* infection may be relatively mild.

**Supplementary Information:**

The online version contains supplementary material available at 10.1186/s13256-023-03818-z.

## Background

*Streptococcus cristatus* (*S. cristatus*) belongs to the Mitis group of the Streptococcus genus. Like many *Streptococcus* species, *S. cristatus* is a commensal bacterium of the human oral cavity. It was first characterized in 1991 [1], as phenetic tests became more sophisticated. One species of oral streptococcus resembled *Streptococcus sanguis*, except with tufts of lateral fibrils. With new phenetic tests and DNA–DNA hybridization experiments, this species was reliably distinguished from other closely related streptococci. It was named *Streptococcus crista* and first described as “Gram-positive, catalase-negative cocci that are approximately 1 μm in diameter and grow in chains,” along with various biochemical descriptors, e.g., α-hemolytic and arginine metabolizer [1].

More than 30 years later, the pathogenicity of *S. cristatus* remains somewhat of a mystery. It appears to be a rare cause of disease, as there are only five cases in the literature [2–4]. They are summarized in Table 1. One other report referred to a patient’s history of “recurrent *Streptococcus cristatus* bacteremia originating from poor dentition” [5] but did not elaborate further.

**Table 1:** Summary of current literature

Study	Patient profile	Site of isolation	Coinfectants	Complications	Antibiotic treatment
Matthys, 2006 [2]	·37-year-old immunocompetent male	Blood	*Streptococcus mitis*	·Cardiac vegetations ·Severe aortic insufficiency requiring prosthetic valve	Ampicillin 6 × 2 g/day and gentamycin 3 × 1.5 mg/kg/day
Matthys, 2006	·52-year-old-male with a history of epilepsy	Resected aortic valve	Coagulase-negative staphylococcus	·Severe aortic insufficiency · Severe mitral insufficiency ·Mitral perforation · Severe left ventricular dilatation ·Left-sided heart failure	·Failed: ceftriaxone and metronidazole ·Succeeded: ampicillin 6 × 2 g/day, gentamycin 3 × 60 mg/day, piperacillin-tazobactam 4 × 4 g/day, and oxacillin 2 g/day
Matthys, 2006	·3-year-old female with a history of mental retardation and epilepsy	Blood	–	–	Amoxicillin-clavulanate
Choe, 2021 [3]	·59-year-old woman with history of glaucoma	Vitreous fluid	–	·Left-sided bleb-related endophthalmitis ·Permanent left-sided vision loss	·Topicals: Tobramycin (2.0%, 20 mg/mL), cefazolin (10%, 100 mg/mL), and moxifloxacin (0.5%, 5.45 mg/mL) ·Systemic: Cefuroxime 1.5 g/day and tobramycin 160 mg/day
Gupta, 2020 [4]	·15-day-old healthy male	Synovial fluid	–	–	Vancomycin

The reports suggest *S. cristatus* can cause infective endocarditis (IE) with major complications, even in healthy adults [2, 3]. There are three cases in adults, two of which involved IE with valvular dysfunction of the aortic and mitral valves, requiring valvular surgery in one patient and progressing to heart failure in the other [2]. The third adult case [3] involved postoperative endophthalmitis after glaucoma surgery, suggesting *S. cristatus* may also inhabit the periocular flora and become pathogenic after breach of the globe exterior.

## Case presentation

A 59-year-old African American male with end-stage cryptogenic cirrhosis presented to the emergency room (ER) endorsing progressive fatigue and confusion for 4 days. At baseline, the patient could complete all activities of daily living but lived with three family members who helped him with instrumental activities of daily living such as cooking and shopping. Per his niece who had accompanied him to the ER, he was now rarely leaving his bed due to fatigue, and his confusion had progressed such that he was now answering basic questions incorrectly.

His cirrhosis had been characterized as cryptogenic after a transplant evaluation failed to reveal an etiology. It had been classified as “end-stage” due to a Model for End-stage Liver Disease (MELD-Na) score of 27 (estimated 90-day mortality of 20%) [6] and a Class C Child–Pugh Score (life expectancy 1–3 years) [7]. He required monthly paracenteses and, in the past, required treatment for spontaneous bacterial peritonitis (SBP), hepatic encephalopathy, and gastrointestinal bleeds (GIBs) secondary to esophageal varices and portal hypertensive gastropathy.

In the ER, he was noted to be somnolent and oriented only to self. A physical exam revealed a distended abdomen with shifting dullness, and a lower extremity exam revealed +2 pitting edema. Labs were remarkable for ammonia of 106 µmol/L and a hemoglobin of 7.0 g/dL, down from a baseline of ~ 9 g/dL. He denied melena or any outright bleeding. Because of his ongoing ascites and altered mental status, he underwent a combined diagnostic/therapeutic paracentesis in the ER. Additionally, two blood cultures were drawn from separate venipuncture sites. He was initiated on treatment for suspected GIB (two large bore intravenous drips, intravenous pantoprazole, intravenous octreotide, intravenous ceftriaxone) and hepatic encephalopathy (lactulose and rifaximin titrated to 3–4 bowel movements per day) and admitted to the general practice unit.

The next day, he underwent esophagogastroduodenoscopy (EGD), which revealed grade one varices and diffuse portal hypertensive gastropathy, but no active bleeding. Octreotide was discontinued at this point. Peritoneal cultures obtained in the ER revealed no evidence of SBP, but both blood cultures grew *Streptococcus cristatus*. Minimal inhibitory concentration using the ETEST method reveal antibiotic susceptibility to ceftriaxone, clindamycin, erythromycin, tetracycline, and vancomycin. The patient had an ongoing history of poor oral hygiene and dental caries, which were likely the source of this bacteremia.

**Fig. 1:**
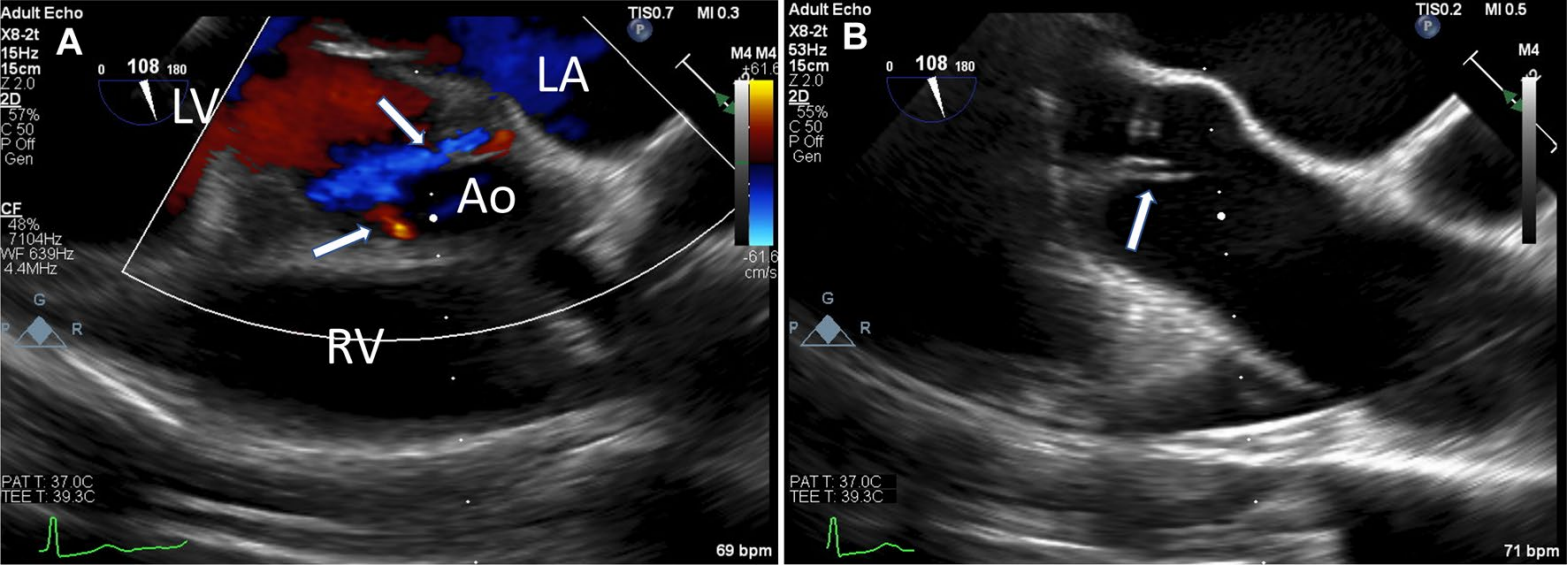
**A** Mid-esophageal transesophageal echocardiogram image displaying two regurgitant jets (arrows) flowing from the aorta (Ao) to the left ventricle (LV), indicative of new onset mild-to-moderate aortic regurgitation; RV, right ventricle; LA, left atrium. **B** A structurally normal aortic valve (arrow) observed in the same view using standard ultrasound mode

Because of his positive blood cultures, we assessed for the presence of IE. We did not appreciate any cardiac murmurs on physical exam or other findings suggestive of IE such as Roth’s spots, Osler’s nodes, or Janeway lesions. However, transthoracic echocardiogram revealed new mild-to-moderate aortic regurgitation compared with prior echocardiograms, without other evidence of IE, in other words no vegetations, abscesses, or perforations. This new-onset aortic regurgitation with positive blood cultures indicated “possible endocarditis” per the modified Duke Criteria [8]. As such, a transesophageal echocardiogram was obtained to further assess for IE. This echocardiogram (Fig. 1) similarly showed mild-to-moderate aortic regurgitation without other findings of IE. Per Duke’s Criteria guidelines, we used clinical judgement at this point and elected not to treat him for IE.

He was treated for bacteremia with a two-week course of cephalosporins. He received 8 days of 1 g/day intravenous ceftriaxone, which was started on day one for prophylactic treatment of suspected upper GIB. Upon discharge, he was transitioned to 400 mg/day of oral cefpodoxime for 6 days. Despite his advanced comorbidities and ongoing GIB, the infection was mild, and he recovered well. He did not exhibit any constitutional symptoms or any Systemic Inflammatory Response Syndrome (SIRS) criteria throughout his entire hospital stay. His mentation improved after lactulose, and subsequent blood cultures remained negative. The infection did not delay his discharge, which was determined primarily by the stability of his hemoglobin. His clinical course is summarized in Table 2.

**Table 2:** Summary of the clinical case

Day	Signs and symptoms	Remarkable labs	Diagnostic tests	Therapeutic interventions
1	Fatigue Confusion Denies constitutional symptoms Denies melena or hemoptysis	·Complete blood count: Hgb 7.0 g/dL, PLT 94,000 per microliter, WBC 4.8 × 10^9^/L ·Liver profile: TBil 3.3 mg/dL, DBil 1.3 mg/dL, ALP 323 IU/L, AST 21 U/L, ALT 8 U/L ·Ammonia: 106 µ/dL ·Urinalysis: Clear ·COVID-19 PCR: Not detected	·CT head: No acute processes · Chest X-ray: No acute process ·Blood culture: *Streptococcus cristatus* × 2 ·Peritoneal culture: no growth	·Therapeutic/diagnostic paracentesis ·Started on lactulose and rifaximin ·Started on octreotide, pantoprazole, and ceftriaxone
2	Fatigue and confusion improved Denies constitutional symptoms Denies melena or hemoptysis	·Hgb 5.9 g/dL pretransfusion ·Hbg 6.9 g/dL post-transfusion	·EGD: no overt signs of bleeding; diffuse and friable portal hypertensive gastropathy	·Ceftriaxone continued for treatment of *S. cristatus* bacteremia ·1 unit of pRBCs ·Octreotide discontinued
3	No complaints Denies melena or hemoptysis	·Hgb: 6.4 g/dL pretransfusion ·Hbg 8.6 g/dL post-transfusion	·TTE: New mild-to-moderate aortic regurgitation but no other evidence of infective endocarditis	·1 unit of pRBCs
6	No complaints	·Hgb 8.3 g/dL	·TEE: similar findings to TTE	
8	Discharged	·Hgb 8.6 g/dL		·Switched to oral cefpodoxime 200 mg

*Hgb* Hemoglobin; *PLT* Platelets; *WBC* White Blood Cells; *TBil* Total Bilirubin; *DBil* Direct Bilirubin; *ALP* Alkaline Phosphatase; *AST* Aspartate Aminotransferase; *ALT* Alanine Aminotransferase; *PCR* Polymerase Chain Reaction; *EGD* Esophagogastroduodenostomy; *pRBC* packed Red Blood Cells; *TTE* Transthoracic Echocardiogram; *TEE* Transesophageal Echocardiogram

## Microbiology

The identification process started with the preparation of blood, MacConkey (Mac), and chocolate agar plates. Each plate was cultured and incubated overnight at 35 ± 2 °C. The following day, alpha-hemolytic growth was seen in the blood and chocolate agar, but not in the Mac agar. The colonies were also found to be catalase-negative, and Gram staining revealed Gram-positive cocci in pairs.

To determine the presence of *S. cristatus*, MALDI-TOF and Vitek 2 biochemical panels were performed. In addition, the Vogus-Proskauer (VP) test was negative, which was used to rule out *Streptococcus sanginosus*. Since *S. cristatus* is a member of the Mitis/Oralis group of the streptococcal species, the biochemical profile was negative for mannitol, urea hydrolysis, and VP, but variable for arginine hydrolysis, esculin, and sorbitol. No other organism was identified at any point in the culturing process.

## Discussion

To summarize, this was a case of *Streptococcus cristatus* bacteremia and “possible endocarditis” in a 59-year-old male with a history of dental disease and end-stage cryptogenic cirrhosis. To our knowledge, this is the sixth clinical case of *S. cristatus*. As outlined in Table 1 [2–4], the other reports involved cases of bacteremia, infective endocarditis, endophthalmitis, and septic arthritis, and they involved both pediatric and middle-aged patients.

Our case is the only case in an adult in which a likely source was identified for the infection. Our patient had a history of poor oral hygiene and dental caries, and *S. cristatus* is an oral bacterium. Like other members of the streptococcus genus, *S. cristatus* can live in harmony with the healthy oral mucosa but with breaks in this protective layer, it appears capable of entering the blood and causing secondary infections. This highlights the importance of the oral exam, especially in patients with higher comorbidities, as poor oral hygiene is a known risk factor for bacteremia and IE [9].

Our case involved an inconclusive diagnosis of “possible endocarditis” based on the presence of bacteremia with new valvular regurgitation. We elected not to treat him for IE on the basis of clinical judgement. If there was any endocardial involvement, it appeared to be minimal, as the patient was asymptomatic, with normal hemodynamic parameters (aside from chronic hypotension secondary to cirrhosis) and relatively normal cardiac parameters on echocardiograms. As such, we were comfortable with a two-week course for bacteremia. Conversely, there are two cases of IE in the literature [2], one of which required surgical valve replacement and the other progressing to heart failure. However, unlike our case, these cases involved a second microbe known to cause IE [10], namely Coagulase-negative Staphylococcus and *Streptococcus mitis*. Thus, the endocardial affinity and virulence of *S. cristatus* may be significantly lower than suggested on the basis of these other cases with coinfectants.

Our case is also the only case with a past medical history of severe chronic disease, as our patient was suffering from end-stage liver disease. Thus, there are now reports of *S. cristatus* infections in generally healthy patients, as well as severely ill patients.

Our patient was successfully treated with an antibiotic regimen consisting of 8 days of intravenous ceftriaxone, transitioning to 6 days of oral cefpodoxime. Ceftriaxone was used in one other case [2], and it did not lead to any clinical or biochemical improvement, prompting a switch to a different regimen. It is not clear from this case whether cephalosporins failed because of progression of *S. cristatus* or the coagulase-negative staphylococcus.

The main limitation of our case was the inconclusive diagnosis of “possible endocarditis.” Isolating *S. cristatus* from our patient’s endocardium, as was done in one previous case [2], could have established a more conclusive result, but our patient did not require valvular surgery. Further reports of isolated *S. cristatus* IE, especially those requiring valvular surgery, could help elucidate *S. cristatus*’s pathogenicity toward the endocardium. Similarly, it would also have been useful to isolate *S. cristatus* from our patient’s oral cavity to directly link his dental disease to the bacteremia.

## Conclusion

A patient with dental disease developed bacteremia and “possible endocarditis” with an oral bacterium called *Streptococcus cristatus*. Whereas other case reports in literature involved severe infective endocarditis, our patient’s infection was mild and was adequately treated with cephalosporin antibiotics.

## Supplementary Information

**Additional file 1.** Video of the transesophageal echocardiogram from Figure 1, showing new-onset mild-tomoderate aortic regurgitation.

## Data Availability

Data sharing is not applicable to this article as no data sets were generated or analyzed during this study.

## Abbreviations

ER: Emergency room
GIB: Gastrointestinal bleed
IE: Infective endocarditis
MELD: Model for End-Stage Liver Disease
SBP: Spontaneous bacterial peritonitis
SIRS: Systemic inflammatory response syndrome
pRBCs: Packed red blood cells
VP: Vogus–Proskauer
